# The opioid industry's use of scientific evidence to advance claims about prescription opioid safety and effectiveness

**DOI:** 10.1093/haschl/qxae119

**Published:** 2024-10-24

**Authors:** Ravi Gupta, Jason Chernesky, Anna Lembke, David Michaels, Cecilia Tomori, Jeremy A Greene, G Caleb Alexander, Adam D Koon

**Affiliations:** Division of General Internal Medicine, Johns Hopkins University School of Medicine, Baltimore, MD, 21287, United States; Department of Health Policy and Management, Bloomberg School of Public Health, Johns Hopkins University, Baltimore, MD, 21205, United States; Department of the History of Medicine and Center for Medical Humanities and Social Medicine, Johns Hopkins University School of Medicine, Baltimore, MD, 21205, United States; Department of Psychiatry and Behavioral Sciences, Stanford University School of Medicine, Stanford, CA, 94304, United States; Milken Institute School of Public Health, George Washington University, Washington, DC, 20037, United States; Johns Hopkins School of Nursing and Bloomberg School of Public Health, Johns Hopkins University, Baltimore, MD, 21205, United States; Division of General Internal Medicine, Johns Hopkins University School of Medicine, Baltimore, MD, 21287, United States; Department of the History of Medicine and Center for Medical Humanities and Social Medicine, Johns Hopkins University School of Medicine, Baltimore, MD, 21205, United States; Center for Drug Safety and Effectiveness, Johns Hopkins Bloomberg School of Public Health, Baltimore, MD, 21205, United States; Center for Drug Safety and Effectiveness, Johns Hopkins Bloomberg School of Public Health, Baltimore, MD, 21205, United States; Department of Epidemiology, Johns Hopkins Bloomberg School of Public Health, Baltimore, MD, 21205, United States; School of Health, Department of Global Health, Georgetown University, Washington, DC, 20057, United States

**Keywords:** opioids, pharmaceuticals, commercial determinants, public health

## Abstract

It is widely recognized that pharmaceutical marketing contributed to the ongoing US opioid epidemic, but less is understood about how the opioid industry used scientific evidence to generate product demand, shape opioid regulation, and change clinician behavior. In this qualitative study, we characterize select scientific articles used by industry to support safety and effectiveness claims and use a novel database, the Opioid Industry Documents Archive, to determine notable elements of industry and non-industry documents citing the scientific articles to advance each claim. We found that 15 scientific articles were collectively mentioned in 3666 documents supporting 5 common, inaccurate claims: opioids are effective for treatment of chronic, non-cancer pain; opioids are “rarely” addictive; “pseudo-addiction” is due to inadequate pain management; no opioid dose is too high; and screening tools can identify those at risk of developing addiction. The articles contributed to the eventual normalization of these claims by symbolically associating the claims with scientific evidence, building credibility, expanding and diversifying audiences and the parties asserting the claims, and obfuscating conflicts of interest. These findings have implications for regulators of industry products and corporate activity and can inform efforts to prevent similar public health crises.

## Introduction

The responsibility of different parties for the ongoing US opioid crisis has been heavily scrutinized. Pharmaceutical manufacturers, for example, have fueled prescription opioid oversupply by targeting clinicians, promoting key opinion leaders, and shaping continuing medical education (CME) and the activities of health professional pain committees, advocacy organizations, and accreditation organizations.^1,2^ Similarly, shortcomings in the pharmaceutical supply chain, including insufficient monitoring, supervision, and risk mitigation, have also contributed to the crisis.^3-7^

It is less well understood how pharmaceutical manufacturers, distributors, retailers, and management consultants (hereafter, “opioid industry”) used scientific evidence to further their commercial interests. While misrepresentation of select evidence, such as a 1980 letter to the editor by Porter and Jick,^8^ has been well documented, this was not the only scientific publication intended to sway professional opinion. Little has been done to place Porter and Jick's publication in the context of similar work that helped fuel the 4-fold increase in prescription opioid use in the US between 1999 and 2011.^9^ This is important because for-profit entities, including, for example, tobacco, fossil fuel, and other pharmaceutical and medical device industries, have a long history of promoting their commercial interests at the expense of public health.^10,11^ Across such industries, a key strategy has been to promote or protect product sales by advancing and normalizing unproven or incorrect scientific claims.^12,13^ Deliberate misrepresentation of evidence in a biased and highly selective manner, such as through exaggeration and promotion of weak evidence and distortion of strong evidence, as well as by casting uncertainty on unfavorable findings, has been described as “spin” or “manufacturing of doubt.”^14-16^ Shaping scientific knowledge and information available to consumers as well as clinicians who prescribe pharmaceutical products like opioids is a key instrument of corporate political activity and influence.^17,18^

To address this gap, we examined how the opioid industry used scientific studies to promote and shape claims about opioid safety and effectiveness. Understanding the opioid industry's use of scientific evidence can sharpen clinicians’ ability to discern reliable information as they make treatment decisions for patients, engage in CME activities, and join professional societies. Moreover, this knowledge can help inform policymakers as they seek to prevent similar industry-driven public health crises.

## Data and methods

### Study design and overview

In this qualitative study, we identified 5 claims that were among the most substantive advanced by industry about the safety and effectiveness of opioids and key scientific articles supporting the claims. We reviewed opioid industry documents using established methods from prior research on tobacco industry documents.^19,20^ Using these methods, we determined the key types of documents citing the scientific articles to support each claim and examined elements of how industry used the articles to advance the claims. This study, which used public, nonidentifiable data, was exempt from institutional review board review. Our data collection, qualitative analysis, and writing process followed the Standards for Reporting Qualitative Research reporting guideline.

### Selection of claims

Opioid industry claims were identified from review of published literature and based on expertise from the authors and an additional expert in clinical medicine and the opioid crisis to ensure that the opioid industry's most consequential claims were represented.^1,21,22^ These were among the most widely advanced claims.

### Evidentiary basis for the opioid industry's claims

To identify peer-reviewed scientific articles supporting each claim, we conducted a targeted search of peer-reviewed literature and publicly available documents. The search of peer-reviewed literature was performed using key words from each claim (eg, “pseudoaddiction”, “opioid screening tools”). Relevant articles were used in snowball searches to identify additional articles—both papers cited by and papers citing the original article in MEDLINE and Google Scholar—pertinent to each claim until saturation was achieved (eTable 1).

### Use of scientific evidence to support claims

To define the key ways that each scientific article was used to support each claim, we performed a thematic analysis using the Opioid Industry Documents Archive (OIDA). The archive, a collaboration between the University of California, San Francisco, and Johns Hopkins University,^23^ is a living, digital repository of millions of documents that have been publicly disclosed through litigation with opioid manufacturers, distributors, pharmacies, and management consultants.^24^

To examine how the opioid industry used scientific articles to advance each claim, 3 investigators (R.G., J.C., A.D.K.) used each article's full title to search documents from OIDA in July 2023 across all included settlements. We extracted, reviewed, and categorized relevant documents (eMethods). We used an OIDA filter to exclude duplicates. Two researchers independently performed each OIDA search and coded source material in duplicate into distinct categories, meeting frequently to review findings and address discrepancies in categorization. A total of 3666 documents that cited 15 scientific papers were identified from OIDA and coded into 6 different types of source documents.

We then iteratively cycled between source data and interpretation to develop themes of how scientific articles were used in industry documents to advance claims, with recurrent internal reflection on the process of generating consistent meaning.^25^ These methods were consistent with prior studies of tobacco,^26^ cannabis,^27^ and pharmaceutical^28,29^ industry documents. Three authors (R.G., J.C., A.D.K.) met frequently to discuss findings, assess disagreements, and reach consensus. Methods for organization and interpretation of the information and core themes pertinent to each article and claim were consistent with logics of abductive inquiry in interpretive policy research^25^ and akin to constant comparative methods in grounded theory.^30^

## Results

We identified 15 scientific articles supporting 5 claims advanced by the opioid industry. Table 1 lists these claims and articles, with representative quotations of how the articles were used to support the claims. The scientific articles were published between 1980 and 2011 and included letters to the editor, case reports, commentaries, original research, and reviews (eTable 2 and eFigure). Most articles were published in internal medicine (*n* = 3) and pain journals (*n* = 10), with some published in social science (*n* = 1) or public health journals (*n* = 1). Many of the articles were published by overlapping sets of co-authors.

**Table 1.** Misuse of scientific evidence by the opioid industry to support claims regarding opioid safety and effectiveness.

Industry claim	Selected articles to support claims	Representative quotations from OIDA documents citing selected articles
1. Opioids are effective for the treatment of chronic, non-cancer pain.	France et al^31^; Portenoy and Foley^32^	“Major progress in the understanding of opioid pharmacology has offered to clinicians a wider range of indications for these drugs, especially in noncancer pain.” (Position paper in the journal *Pain Medicine*).
2. Opioids are “rarely” addictive as long as they are prescribed by a doctor to a patient with pain.	Perry and Heidrich^33^; Porter and Jick^8^; Fishbain et al^34^; Joranson et al^35^; Fishbain et al^36^	“The authors found that the present trend of increasing medical use of opioid analgesics to treat pain does not appear to contribute to opioid analgesic abuse. This article should be helpful in discussing these issues with your physicians and should enhance your understanding of the potential for abuse or misuse in the context of pain management.” (Email from Medical Services, Product Management, Sales Training, Janssen Pharmaceuticals to Sales Force)
3. Individuals who appear to be developing addiction to prescription opioids from a doctor may have “pseudoaddiction,” requiring more opioids to treat what is actually continued pain.	Weissman and Haddox^37^; McQuay^38^; Fishbain^39^	“Challenges in diagnosis: Pseudoaddiction—behavior with characteristics of addition but rational, considering the patient's situation (eg, inadequately treated chronic pain patients. May also apply to some patients who “self treat” underlying mental health disorders). (See Weissman 1989.) *Pseudoaddiction should be ruled out before making a substance use disorder diagnosis*.” (2014 Health Care for the Homeless Clinicians’ Network, Recommendations for the Care of Homeless Patients with Opioid Use Disorders)
4. No dose is too high, and if a patient develops tolerance to opioids, the solution may be to further increase the dose to treat the pain.	Portenoy et al^40^; Foley et al^41^	“Methods developed to identify potential medication misuse. Aberrant behaviors identified by clinicians—Such behaviors include forging prescriptions, stealing or borrowing drugs, frequently losing prescriptions, and resisting changes to medication despite adverse side effects.” (2008 Advances in Pain Management, Continued Medical Education)
5. Screening tools can identify patients who will abuse medications and/or develop addiction to prescription opioids.	Webster and Webster^42^; Belgrade et al^43^; Passik and Kirsh^44^	“To determine a patient's risk of medication abuse, resources such as the Opioid Risk Tool (ORT) may be effective. Together, these tools can help clinicians more thoroughly consider the attributes that make patients appropriate candidates for opioid therapy.” (2014 promotional email from Janssen Pharmaceuticals marketing team for Nucynta targeting physicians)

Abbreviation: OIDA, Opioid Industry Documents Archive.

### OIDA document types using the selected scientific articles

Six types of source materials were generated from review of OIDA documents referencing the scientific articles: teaching or training materials, knowledge-brokering materials, articles or textbooks, regulatory filings, emails, and other (Table 2). Scientific articles were featured most frequently in other academic articles (often commentaries or editorials), followed by knowledge-brokering materials (eg, CME course modules), teaching or training materials (eg, presentations for industry marketing teams), and emails. Regulatory filings were unusual in that some new drug applications or incident reporting documents listed the relevant scientific articles in a references section, but in-text citations were either missing or ambiguous.

**Table 2.** Use of selected scientific articles within the opioid industry documents archive.

Form of use	Summary
Articles or textbooks	Many of the scientific articles examined were in turn cited in other peer-reviewed publications as well as in trade press or other industry-friendly publications. In several notable examples, pain-management textbooks used the selected scientific articles to support the same claims advanced by the opioid industry.
Emails	The selected articles were noted in emails less commonly than in other types of OIDA documents. Nevertheless, the articles were included in emails to sales force teams accompanied by strategies for how to incorporate them into conversations with prescribers.
Knowledge brokering	A large number of articles were used to support claims in documents tailored for a wide variety of audiences. The articles were included in continuing medical education course modules targeting physicians and nurses, most of which were either sponsored or supported by educational grants from various firms in the opioid industry. Articles were used in other documents, including organization consensus statements, professional society guidelines and summative reports, pamphlets, websites launched by opioid industry firms targeting clinicians and patients with information, and other advertising materials, that obscured the boundaries of knowledge dissemination, advocacy, and marketing.
Teaching or training	The selected articles were used in opioid industry presentations to train pharmaceutical detailers and sales force members, medical science liaisons, and key opinion leaders to target health care professionals and alleviate their concerns about prescribing opioids. Other teaching or training materials in which the selected articles were used included reference guides, pamphlets/literature for prescribing health care professionals, and website articles.
Regulatory	The scientific articles were often listed as references in regulatory documents without any in-text citations supporting the specific claims. The majority of regulatory documents including the selected articles were composed of new drug applications submitted to the FDA for opioid products, such as Exalgo (hydromorphone); product monographs detailing drug characteristics such as indications and clinical use, contraindications, and warnings and precautions; and documents stating post-marketing surveillance strategies or findings.
Other	Many of the scientific articles were included in a variety of documents that arose directly from litigation, such as testimonies from individuals or firms. In several cases, firms from the opioid industry were asked to respond directly to the specific claims included in this study and the scientific articles used to support the claims.

Abbreviations: FDA, US Food and Drug Administration; OIDA, Opioid Industry Documents Archive.

### Use of scientific articles to support opioid prescribing

**Figure 1.**
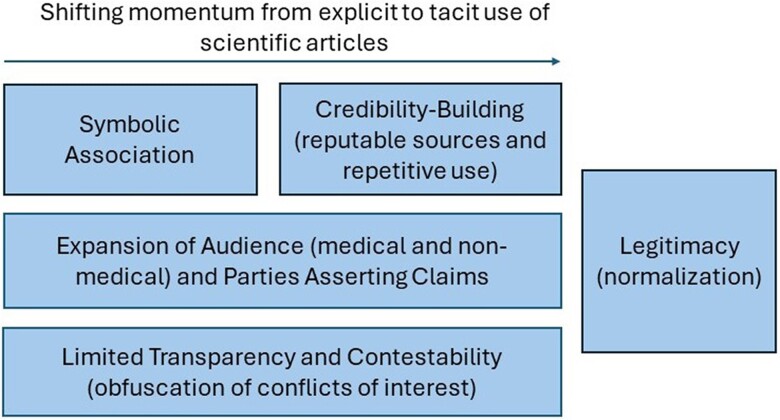
Key elements of the opioid industry's use of scientific articles to advance claims. Several elements were derived from review of the opioid industry documents about the misuse of scientific evidence to advance claims. Symbolic association, credibility-building, expansion of audiences and parties asserting the claims, and limited transparency and contestability allowed the legitimization of scientific claims. These elements reflected a shifting momentum from explicit to tacit use of the scientific articles.

Review of the OIDA documents generated 4 key elements of how the scientific articles were used: (1) symbolic association of the claims with the scientific evidence, (2) credibility-building of the claims through repeated use of the knowledge base, (3) expansion and diversification of the audience to whom the claims were directed and the parties asserting the claims, and (4) limited transparency and contestability of the claims through obfuscation of conflicts of interest. Figure 1 illustrates how these elements reflected a shifting momentum from explicit to tacit use of the scientific articles. The uses were not limited to industry—non-industry organizations frequently advanced the same claims and cited the same scientific articles.

#### Symbolic association

In regulatory documents submitted by the opioid industry to the Food and Drug Administration (FDA), including new drug applications, scientific articles were included in the references section without any in-text citations. For example, in an amendment to a new drug application submitted to the FDA for Roxicodone immediate-release tablets,^45^ citations including Porter 1980, Portenoy 1986, Portenoy 1990, and McQuay 1999 were listed without being discussed in the application itself. In this way, the articles lent a sense of scientific expertise and validity to the claims within the applications simply by being cited. Industry documents revealed how the scientific articles were used symbolically and in a performative manner to support the opioid industry's claims. By leveraging a shared vocabulary placed in a familiar context, claims were assumed to be fact, without compelling more thorough interrogation of the articles themselves or the rigor of the methods used, and often, without a direct citation to the article.

#### Credibility-building

**Figure 2.**
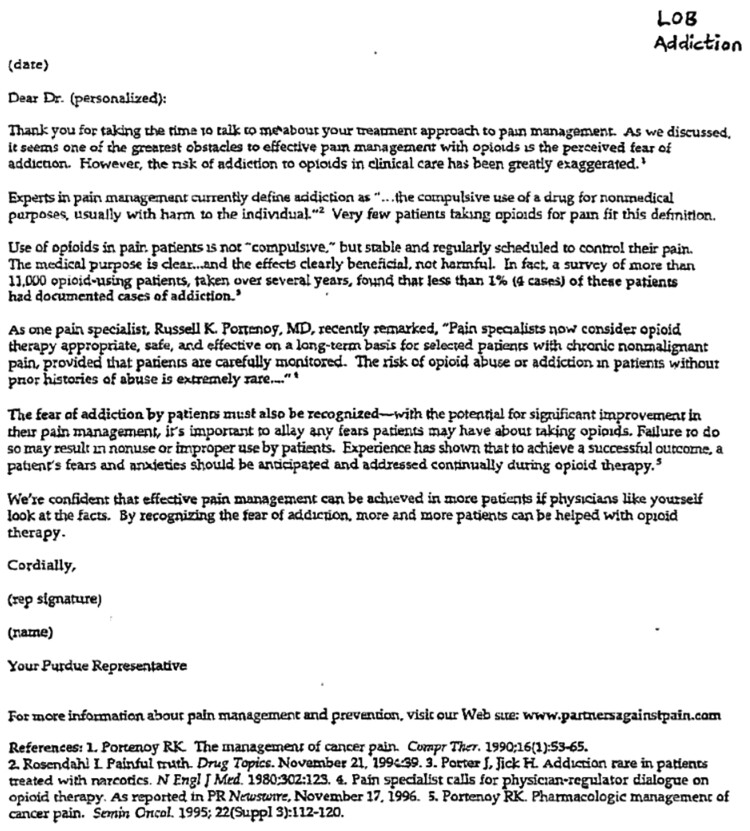
Purdue sales force template to respond to prescriber concerns about opioid addiction.^46^ This letter, included in a document identified from the Opioid Industry Documents Archive as part of the National Prescription Opiate Litigation Documents, provided sales force team members with a template to respond to physicians’ concerns about the risk of addiction from opioids. The letter mischaracterized a 1980 1-paragraph letter to the editor that described the rate of addiction among hospitalized patients at an unknown follow-up time.

In industry presentations to sales force members who visited physicians to encourage them to prescribe opioids, the scientific articles were used to make claims about the rarity of addiction. The findings of the underlying studies, however, were exaggerated or incorrectly extrapolated to unrelated settings, such as findings from a study of addiction during treatment of acute pain being applied to chronic pain. For instance, Purdue Pharmaceuticals sent template letters to its sales force team with marketing tips for communicating about OxyContin to physicians. One letter responding to prescriber concerns about addiction cited Porter et al and mischaracterized the duration of treatment with opioids in the study **(**Figure 2).^46^ The documents revealed how repeatedly advancing scientific claims supported by recognized experts in the emerging field of pain management built confidence among various audiences about the validity of the claims. A network of reputable individuals, including practitioners, researchers, and advocates with an authoritative claim to pain-specific knowledge, were deemed to be reliable arbiters and brokers of scientific knowledge. Thus, scientific articles lent credibility to individual claims despite the fact that, in many cases, OIDA documents revealed inaccurate statements contradicting the actual methods or conclusions of the underlying scientific articles.

#### Expansion of audience and parties asserting claims

A wide array of CME activities supported by education grants from the opioid industry cited the scientific articles. For example, a CME activity sponsored by several pharmaceutical companies and targeting primary care physicians and pain specialists cited Webster and Webster^42^ and stressed the importance of using the Opioid Risk Tool, a screening tool designed to identify patients’ likelihood of subsequent abuse or misuse when prescribing opioids for chronic non-cancer pain.^47^ Such screening tools appeared to have limited efficacy in identifying patients at risk of developing opioid addiction.^48,49^ Documents revealed how the scientific articles were used to support industry claims to target a diverse and expanding spectrum of audiences, including prescribing physicians, foundations, patient advocacy groups, and regulators. In certain instances, the uses of the scientific articles were explicit, such as in presentations to sales force members who could use the scientific evidence to persuade prescribing health care professionals. In other instances, the articles were used tacitly, such as citations in official guidelines and consensus statements from trusted institutions and professional societies.

Moreover, non-industry organizations, such as nonprofit, civic, or governmental organizations, began relying on the scientific evidence and advancing the same claims. Recommendations from the Healthcare for the Homeless Clinicians’ Network, for example, listed “pseudoaddiction” as a challenge to diagnosing opioid use disorder, writing, “Pseudoaddiction should be ruled out before making a substance use disorder diagnosis.”^50^ The recommendations cited Weissman and Haddox,^37^ a case report of a single patient upon which the concept was created, to support this statement. Although the organization may have been unaware of the shortcomings of this scientific article and of the absence of any subsequent empirical studies validating the concept, their use of the article reflected the growing legitimacy of the claims and the increasing body of literature supporting the claim.

#### Obfuscating conflicts of interest

A number of selected articles were authored by experts, particularly in the field of pain management, who served as consultants or advisory board members for key firms in the opioid industry, sometimes concurrently while conducting the studies and writing the articles. For example, Drs. Portenoy, Passik, Fishbain, Webster, Belgrade, and Mr. Joranson, all first or senior authors of key selected articles, each served at some point as consultants, grantees, or advisory board members for the opioid industry. Moreover, many conflicts appeared to be missing: for example, Dr. Fishbain served as an expert witness for Purdue Pharmaceuticals prior to 2009,^51^ but this conflict was not disclosed in his 2008 article downplaying the addictiveness of opioids.^36^ In other cases, there were similar conflicts among individuals, like Dr. Webster, who in 2013 simultaneously served as a key opinion leader for Mallinckrodt and as president of the American Academy of Pain Medicine, where he authored treatment guidelines promoting opioid screening tools.^52^ In addition, aside from eventual consulting and advisory board service, much of Dr. Portenoy's research in the 1980s and 1990s was funded by financial support from pharmaceutical companies.^53^ The lack of transparency was important in limiting readers’ ability to assess how underlying motivations or intentions could shape scientific judgment.

## Discussion

Our thematic analysis of documents from select opioid settlements demonstrates that several claims advanced by the opioid industry about the safety and effectiveness of prescription opioids were supported by misrepresentation of a scientific foundation. Documents suggested that these scientific articles helped contribute to the normalization of claims about prescription opioids, seeking to influence professional norms in the US around pain treatment, which, due to the very real problem of limited resources for patients with pain, may have allowed for greater clinician, policymaker, and patient receptiveness to the claims. These competencies, supported by enormous corporate resources, demonstrated a remarkable capacity for industry to profoundly and tacitly shape the information environment and clinical prescribing behavior for opioids in the early 2000s.

This study extends existing literature examining the factors that have driven the opioid crisis and advances efforts to curtail the misrepresentation of scientific evidence for commercial benefit in several ways. Prior investigations have focused on a narrow set of opioid industry claims from a limited number of actors, including Purdue,^22^ mentioning a few notable supporting scientific articles, such as the Porter and Jick letter and articles authored by Portenoy.^1,21,54^ We built on this work by defining a broader set of industry claims and identifying additional supporting scientific articles. Our analysis is also the first to use novel archival material from OIDA to construct a more comprehensive narrative of industry's misrepresentation of individual scientific articles to attempt to shape clinician behavior. Finally, we extended prior methods used to study the tobacco archives,^19,20,55,56^ utilizing multiple sources and linking peer-reviewed literature to industry documents.

A key finding of our analysis was the shift in momentum from explicit to tacit use of the scientific articles, reflecting a transition from the symbolic use of scientific articles to the normalization of opioid industry claims and the scientific evidence underlying their claims. Furthermore, by repeatedly advancing the studies and the claims in a wide array of documents to an expanding audience and paying the authors of many articles before, during, or after they authored the articles, the opioid industry appeared to build a veneer of scientific legitimacy, allowing it to disarm criticism and dispel doubt regarding their claims. The widespread acceptance of the scientific evidence and the claims was revealed in the documents: while industry often included the scientific articles in sales force trainings, CME activities, and regulatory documents to the FDA to support their claims, the same articles and claims were also repeated by other academics, civic organizations, and medical professional committees. These actions, while likely inadvertent, further reified and made more pervasive the unsubstantiated claims and the validity of the scientific articles supporting the claims. Normalization of the claims also made them more difficult to challenge or undo. Together, these 2 mutually reinforcing elements of normalization and obfuscation allowed the use of inaccurate claims about prescription opioids to drive aggressive marketing tactics.^29,57^

The large number of examined documents targeting clinicians highlights the opioid industry's well-known priority of shaping clinician prescribing behavior. Opioids differ from products like tobacco, alcohol, and unhealthy and ultra-processed foods in that prescription opioids are pharmaceutical products approved by regulatory agencies and prescribed by clinicians for the treatment of pain. While the present findings focus on the misuse of specific scientific articles, others have written on coinciding forms of shaping clinician knowledge and increasing comfort with prescribing opioids, such as through the use of key opinion leaders, advisory boards, and speaker programs; industry-supported ghostwriting of published articles^28,58^; and targeting marketing efforts towards high prescribers of opioids.^29,59^ Clinicians’ role in the opioid crisis and their receptiveness to industry's claims thus has important consequences for CME, participation in professional societies, consumption of scientific evidence, and interaction with industry, particularly as they balance adequately treating pain. Individual clinicians may have limited power in constructing stronger guardrails required for publication of scientific evidence, conflicts of interest, and interactions with industry through consulting and participation as speakers. However, increased awareness of how industry sponsors and interprets scientific evidence about pharmaceuticals like opioids, while minimizing industry interactions that may create conflicts of interest, is an important step.^60^ Support from health systems and medical societies for alternative forms of medical education that are free of commercial bias, such as academic detailing, could help mitigate industry influence over clinical prescribing behavior.^61,62^

Our findings suggest that the opioid industry's strategies to use and misuse science followed a playbook across a number of industries selling products that directly or indirectly impact health, including tobacco, food and drink, alcohol, fossil fuels, pharmaceutical products and medical devices, and gambling.^63^ The tobacco industry pioneered this set of strategies, developing a campaign to promote smoking and sow doubt about the associations between tobacco use and lung cancer. The tobacco industry engineered the public's and the medical community's understanding of this danger^64^ by attempting to discredit epidemiologic studies supporting these findings (which they termed “junk science”), promoting skeptical scientists and funding biased studies that produced contrary evidence, and creating a vast public relations campaign that included establishing the Tobacco Industry Research Committee in 1953.^65-67^ While the tobacco industry focused on changing evidentiary standards for credible science, there was some overlap in their strategies^16^ with those of the opioid industry, including misrepresenting and extrapolating weak or false scientific evidence, expanding and diversifying its target audience, and gaining support from reputable authors to create uncertainty about the risks of their products, disarm criticism, and normalize claims about their products’ benefits.

Our findings have wide-ranging implications for public health, policymakers, and regulators. To build a network of influence across prescribers, regulators, professional committees, and academics, the opioid industry shaped the information environment and misrepresented scientific evidence in pursuit of sales. Their actions’ enormous impact on public health comprises one of several aspects of commercial determinants of health, a concept that connects commercial activities and market forces with their influence on health.^10,68^ While broad political and economic structures enable industry's ability to use and misuse science as a subject and tool of corporate influence, specific tactics used by industries to market and increase sales of harmful products^11^ were seen in our analysis: creating fictitious new diseases (“pseudoaddiction”), information laundering (using questionable sources to mainstream ideas), and astroturfing (obscuring industry sponsorship of article authors and CME activities). In addition, the forms of shaping and wielding scientific knowledge described in this study are common across other industries,^16,69-71^ as synthesized in a recent scoping review of corporate influence over science^72^: skewing publication of science towards industry's favor (limited transparency and contestability), undermining and creating doubt about strong evidence contrary to industry claims and overstating weak evidence supporting their claims (credibility building), manufacturing trust in industry and its claims (credibility building), influencing the reach of science to create an “echo chamber” (expansion of audience and parties asserting claims), and creating industry-friendly policymaking environments (symbolic association). Taken together, these corporate strategies require politically challenging policy and structural responses that limit such commercial influence and stretch beyond simple conflict-of-interest policies.^63,73^

### Limitations

Our study has limitations. First, the OIDA is a dynamic collection of millions of industry records with new documents continually added upon release from ongoing opioid settlements. Thus, our study draws conclusions from a snapshot of records available at the time of our search. Second, a notable exclusion from our study are documents from the Purdue settlement. The Purdue bankruptcy plan and, as a result, plans for document disclosure from the settlement, was ongoing at the time of our study. However, our study is the first, to our knowledge, to assess the scientific foundation for a wider set of claims advanced by parties beyond Purdue and across the opioid industry. Third, our OIDA search strategy involved article titles, as opposed to author names or concepts (eg, “pseudoaddiction”). As a result, we may have excluded notable documents, although after reviewing nearly 4000 documents, we reached thematic saturation. Fourth, while we used established methods and sought to ensure rigor and transparency, the list of included opioid industry claims and the identified scientific articles used to support those claims are not meant to be exhaustive. Indeed, there were many more scientific articles published supporting the opioid industry's claims. The present study includes several substantive claims advanced by the opioid industry and a set of pivotal scientific articles. Fifth, our methods do not estimate associations between the use of scientific evidence and clinical prescribing. While most of the scientific articles included in this study were published prior to surges in prescription opioids, some articles were published later. Nor are we able to directly link the timing of industry payments to consultants to the publication of all articles. Thus, our results are suggestive that industry's claims relied on scientific evidence to build credibility over time to normalize the claims.

## Conclusion

We found that several substantive claims advanced by the opioid industry regarding the safety and effectiveness of prescription opioids, which together were key drivers of the opioid crisis, gained legitimacy by the misrepresentation of a scientific foundation to disarm criticism and promote opioid sales. This was achieved through symbolically associating industry claims with scientific articles, building credibility by relying on knowledge brokers with underlying conflicts of interest, expanding the target audience and the parties asserting the claims, and limiting transparency. These findings have implications for clinicians as they rely on scientific evidence to base clinical decisions and are members of professional societies as well as policymakers who regulate industry products. The results may inform efforts to prevent similar public health crises.

## Supplementary Material

qxae119_Supplementary_Data

## Data Availability

Study data are available upon request.
