# Supplementary material for: The opioid industry's use of scientific evidence to advance claims about prescription opioid safety and effectiveness
Source: Health Aff Sch. 2024 Oct 24;2(10):qxae119. doi: 10.1093/haschl/qxae119 (PMC11500661; doi:10.1093/haschl/qxae119)
Supplement: qxae119_Supplementary_Data [file qxae119_supplementary_data.zip › opioids_science_supplement.docx]

**Supplemental Online Content**

The Opioid Industry’s Use of Scientific Evidence to Advance Claims about Prescription Opioid Safety and Effectiveness

**eMethods.**

**eReferences.**

**eTable 1. Scientific Articles Supporting Opioid Industry Claims**

**eTable 2. Articles Selected for OIDA Search with Number of Documents Found**

**eFigure. Notable Documents from OIDA Citing Scientific Articles to Support Claims**

This supplemental material has been provided by the authors to give readers additional information about their work.

**eMethods.**

**Search strategy to investigate OIDA documents**

Description of OIDA

The OIDA represents the newest collection of industry records in UCSF’s Industry Document Library, which contains millions of documents from the tobacco, chemical, drug, food, and fossil fuel industries. The OIDA is a living, digital repository of millions of previously confidential corporate documents released as part of select settlements with opioid litigation defendants, including Insys, Mallinckrodt, McKinsey, and Walgreens.^1^ The collections are a rich source of documents, including internal communications, training materials, and sales and audit reports that provide valuable insights into the strategies and operations of the opioid industry in relation to the opioid crisis. OIDA is financially supported by these settlement funds.

Search terms used in OIDA

| **No.** | **Paper Title** | **Search Term in OIDA** |
| --- | --- | --- |
| 1 | France RD, Urban BJ, Keefe FJ. Long-term use of narcotic analgesics in chronic pain. *Soc Sci Med.* 1984;19(12):1379-1382 | “Long-term use of narcotic analgesics in chronic pain” |
| 2 | Portenoy RK, Foley KM. Chronic use of opioid analgesics in non-malignant pain: report of 38 cases. *Pain.* 1986;25(2):171-186 | “Chronic use of opioid analgesics in non-malignant pain: report of 38” |
| 3 | Weissman DE, Haddox DJ. Opioid pseudoaddiction--an iatrogenic syndrome. *Pain.* 1989;36(3):363-366. | “Opioid pseudoaddiction--an iatrogenic syndrome” |
| 4 | McQuay H. Opioids in pain management. *Lancet.* 1999;353(9171):2229-2232 | “Opioids in pain management” McQuay |
| 5 | Fishbain DA. Medico-legal rounds: Medico-legal issues and breaches of "standards of medical care" in opioid tapering for alleged opioid addiction. *Pain Med.* 2002;3(2):135-142; discussion 143-136 | “Medico-legal rounds: Medico-legal issues and breaches of "standards of medical care" in opioid tapering for alleged opioid addiction” |
| 6 | Perry S, Heidrich G. Management of pain during debridement: a survey of U.S. burn units. *Pain.* 1982;13(3):267-280 | “Management of pain during debridement: a survey of U.S. burn units” |
| 7 | Porter J, Jick H. Addiction rare in patients treated with narcotics. *N Engl J Med.* 1980;302(2):123 | “Addiction rare in patients treated with narcotics” |
| 8 | Fishbain DA, Rosomoff HL, Rosomoff RS. Drug abuse, dependence, and addiction in chronic pain patients. *Clin J Pain.* 1992;8(2):77-85 | “Drug abuse, dependence, and addiction in chronic pain patients” |
| 9 | Joranson DE, Ryan KM, Gilson AM, Dahl JL. Trends in medical use and abuse of opioid analgesics. *JAMA.* 2000;283(13):1710-1714. | “Trends in medical use and abuse of opioid analgesics” |
| 10 | Fishbain DA, Cole B, Lewis J, Rosomoff HL, Rosomoff RS. What percentage of chronic nonmalignant pain patients exposed to chronic opioid analgesic therapy develop abuse/addiction and/or aberrant drug-related behaviors? A structured evidence-based review. *Pain Med.* 2008;9(4):444-459 | “What percentage of chronic nonmalignant pain patients exposed to chronic opioid analgesic therapy develop abuse/addiction and/or aberrant drug-related behaviors? A structured evidence-based review” |
| 11 | Portenoy RK, Foley KM, Inturrisi CE. The nature of opioid responsiveness and its implications for neuropathic pain: new hypotheses derived from studies of opioid infusions. *Pain.* 1990;43(3):273-286. | “The nature of opioid responsiveness and its implications for neuropathic pain: new hypotheses derived from studies of opioid infusions” |
| 12 | Foley KM, Fins JJ, Inturrisi CE. A true believer's flawed analysis. *Arch Intern Med.* 2011;171(9):867-868; author reply 868 | “A true believer's flawed analysis” |
| 13 | Webster LR, Webster RM. Predicting aberrant behaviors in opioid-treated patients: preliminary validation of the Opioid Risk Tool. *Pain Med.* 2005;6(6):432-442. | “Predicting aberrant behaviors in opioid-treated patients: preliminary validation of the Opioid Risk Tool” |
| 14 | Belgrade MJ, Schamber CD, Lindgren BR. The DIRE score: predicting outcomes of opioid prescribing for chronic pain. *J Pain.* 2006;7(9):671-681. | “The DIRE score: predicting outcomes of opioid prescribing for chronic pain” |
| 15 | Passik SD, Kirsh KL. An opioid screening instrument: long-term evaluation of the utility of the pain medication questionnaire by Holmes et al. *Pain Pract.* 2006;6(2):69-71. | “An opioid screening instrument: long-term evaluation of the utility of the pain medication questionnaire by Holmes et al” |

**eReferences.**

1. Caleb Alexander G, Mix LA, Choudhury S, et al. The Opioid Industry Documents Archive: A Living Digital Repository. *Am J Public Health.* 2022;112(8):1126-1129.

2. Portenoy RK, Foley KM. Chronic use of opioid analgesics in non-malignant pain: report of 38 cases. *Pain.* 1986;25(2):171-186.

3. Chou R, Fanciullo GJ, Fine PG, et al. Clinical guidelines for the use of chronic opioid therapy in chronic noncancer pain. *J Pain.* 2009;10(2):113-130.

4. France RD, Urban BJ, Keefe FJ. Long-term use of narcotic analgesics in chronic pain. *Soc Sci Med.* 1984;19(12):1379-1382.

5. Urban BJ, France RD, Steinberger EK, Scott DL, Maltbie AA. Long-term use of narcotic/antidepressant medication in the management of phantom limb pain. *Pain.* 1986;24(2):191-196.

6. Caldwell JR, Rapoport RJ, Davis JC, et al. Efficacy and safety of a once-daily morphine formulation in chronic, moderate-to-severe osteoarthritis pain: results from a randomized, placebo-controlled, double-blind trial and an open-label extension trial. *J Pain Symptom Manage.* 2002;23(4):278-291.

7. Meske DS, Lawal OD, Elder H, Langberg V, Paillard F, Katz N. Efficacy of opioids versus placebo in chronic pain: a systematic review and meta-analysis of enriched enrollment randomized withdrawal trials. *J Pain Res.* 2018;11:923-934.

8. Dickinson BD, Altman RD, Nielsen NH, Williams MA. Use of opioids to treat chronic, noncancer pain. *West J Med.* 2000;172(2):107-115.

9. Peloso PM, Bellamy N, Bensen W, et al. Double blind randomized placebo control trial of controlled release codeine in the treatment of osteoarthritis of the hip or knee. *J Rheumatol.* 2000;27(3):764-771.

10. Arkinstall W, Sandler A, Goughnour B, Babul N, Harsanyi Z, Darke AC. Efficacy of controlled-release codeine in chronic non-malignant pain: a randomized, placebo-controlled clinical trial. *Pain.* 1995;62(2):169-178.

11. Harati Y, Gooch C, Swenson M, et al. Maintenance of the long-term effectiveness of tramadol in treatment of the pain of diabetic neuropathy. *J Diabetes Complications.* 2000;14(2):65-70.

12. Roth SH, Fleischmann RM, Burch FX, et al. Around-the-clock, controlled-release oxycodone therapy for osteoarthritis-related pain: placebo-controlled trial and long-term evaluation. *Arch Intern Med.* 2000;160(6):853-860.

13. Porter J, Jick H. Addiction rare in patients treated with narcotics. *N Engl J Med.* 1980;302(2):123.

14. Perry S, Heidrich G. Management of pain during debridement: a survey of U.S. burn units. *Pain.* 1982;13(3):267-280.

15. Fishbain DA, Rosomoff HL, Rosomoff RS. Drug abuse, dependence, and addiction in chronic pain patients. *Clin J Pain.* 1992;8(2):77-85.

16. Fishbain DA, Cole B, Lewis J, Rosomoff HL, Rosomoff RS. What percentage of chronic nonmalignant pain patients exposed to chronic opioid analgesic therapy develop abuse/addiction and/or aberrant drug-related behaviors? A structured evidence-based review. *Pain Med.* 2008;9(4):444-459.

17. Joranson DE, Ryan KM, Gilson AM, Dahl JL. Trends in medical use and abuse of opioid analgesics. *JAMA.* 2000;283(13):1710-1714.

18. Moulin DE, Iezzi A, Amireh R, Sharpe WK, Boyd D, Merskey H. Randomised trial of oral morphine for chronic non-cancer pain. *Lancet.* 1996;347(8995):143-147.

19. Medina JL, Diamond S. Drug dependency in patients with chronic headaches. *Headache.* 1977;17(1):12-14.

20. Higgins C, Smith BH, Matthews K. Incidence of iatrogenic opioid dependence or abuse in patients with pain who were exposed to opioid analgesic therapy: a systematic review and meta-analysis. *Br J Anaesth.* 2018;120(6):1335-1344.

21. Weissman DE, Haddox DJ. Opioid pseudoaddiction--an iatrogenic syndrome. *Pain.* 1989;36(3):363-366.

22. Fishbain DA. Medico-legal rounds: Medico-legal issues and breaches of "standards of medical care" in opioid tapering for alleged opioid addiction. *Pain Med.* 2002;3(2):135-142; discussion 143-136.

23. McQuay H. Opioids in pain management. *Lancet.* 1999;353(9171):2229-2232.

24. Portenoy RK, Foley KM, Inturrisi CE. The nature of opioid responsiveness and its implications for neuropathic pain: new hypotheses derived from studies of opioid infusions. *Pain.* 1990;43(3):273-286.

25. Foley KM, Fins JJ, Inturrisi CE. A true believer's flawed analysis. *Arch Intern Med.* 2011;171(9):867-868; author reply 868.

26. Belgrade MJ, Schamber CD, Lindgren BR. The DIRE score: predicting outcomes of opioid prescribing for chronic pain. *J Pain.* 2006;7(9):671-681.

27. Webster LR, Webster RM. Predicting aberrant behaviors in opioid-treated patients: preliminary validation of the Opioid Risk Tool. *Pain Med.* 2005;6(6):432-442.

28. Passik SD, Kirsh KL. An opioid screening instrument: long-term evaluation of the utility of the pain medication questionnaire by Holmes et al. *Pain Pract.* 2006;6(2):69-71.

**eTable 1. Scientific Articles Supporting Opioid Industry Claims**

| **Opioid Industry Claim** | **Most Cited Peer-Reviewed Articles (MEDLINE, Google Scholar citations)**^a^ |
| --- | --- |
| **Claim 1:** Opioids are effective for the treatment of chronic, non-cancer pain. | 1. Portenoy 1986 (149, 1179)^2^ ^b^ 2. Chou 2009 (2543, 640)^3^ 3. France 1984 (13, 162)^4^ ^b^ 4. Urban 1986 (13, 176)^5^ 5. Caldwell 2002 (44, 293)^6^ 6. Meske 2018 (29, 56)^7^ 7. Dickinson 2000 (3, 32)^8^ 8. Peloso 2000 (37, 226)^9^ 9. Arkinstall 1995 (27, 227)^10^ 10. Harati 2000^11^ 11. Roth 2000^12^ |
| **Claim 2:** Opioids are ‘rarely’ addictive as long as they are being prescribed by a doctor to a patient with pain. | 1. Porter/Jick 1980 (174, 1538)^13^ ^b^ 2. Perry/Heidrich 1982 (27, 484)^14^ ^b^ 3. Fishbain 1992 (59, 444)^15^ ^b^ 4. Fishbain 2008 (142, 664)^16^ ^b^ 5. Joranson 2000 (101, 662)^17^ ^b^ 6. Moulin 1996 (62, 539)^18^ 7. Medina 1976 (7, 133)^19^ 8. Higgins 2018 (30, 90)^20^ |
| **Claim 3:** Individuals who appear to be getting addicted to prescription opioids from a doctor have “pseudoaddiction” and are in pain, requiring more opioids. | 1. Weissman and Haddox 1989 (93, 809)^21^ ^b^ 2. Fishbain 2002 (455, 16)^22 b,c^ 3. McQuay 1999 (71, 539)^23 b^ |
| **Claim 4:** No dose is too high, and if a patient develops tolerance to opioids, the solution may be to further increase the dose to treat the pain. | 1. Portenoy 1990 (60, 739)^24^ ^b^ 2. Foley 2011 (2, 8)^25^ ^b,c^ |
| **Claim 5:** Screening tools can predict who will get addicted to prescription opioid. | 1. Belgrade 2006 (38, 229)^26^ ^b^ 2. Webster 2005 (253, 1214)^27^ ^b^ 3. Passik 2006 (4, 8)^28^ ^b^ |

Abbreviations: OIDA, Opioid Industry Documents Archive

^a^ We used Google Scholar and MEDLINE to determine the number of times each article was cited for an initial approximation of its use both in the grey literature and peer-reviewed literature to supplement expert review in identifying the scientific articles used by the opioid industry to support its claims.

^b^ While the table includes all representative articles employed by the opioid industry to support individual claims about opioids that we used to search OIDA, these specific articles were selected to further investigate the opioid industry’s method of use in OIDA.

^c^ Article was used in very few documents or no relevant documents in the OIDA search.

**eTable 2. Articles Selected for OIDA Search with Number of Documents Found^a^**

| **No.** | **Paper Title** | **Claim** | **Number of Documents Found in OIDA** |
| --- | --- | --- | --- |
| 1 | France RD, Urban BJ, Keefe FJ. Long-term use of narcotic analgesics in chronic pain. *Soc Sci Med.* 1984;19(12):1379-1382 | Effective for chronic, non-cancer pain | 43 |
| 2 | Portenoy RK, Foley KM. Chronic use of opioid analgesics in non-malignant pain: report of 38 cases. *Pain.* 1986;25(2):171-186 | Effective for chronic, non-cancer pain | 156 |
| 3 | Perry S, Heidrich G. Management of pain during debridement: a survey of U.S. burn units. *Pain.* 1982;13(3):267-280 | Rarely addictive | 37 |
| 4 | Porter J, Jick H. Addiction rare in patients treated with narcotics. *N Engl J Med.* 1980;302(2):123 | Rarely addictive | 258 |
| 5 | Fishbain DA, Rosomoff HL, Rosomoff RS. Drug abuse, dependence, and addiction in chronic pain patients. *Clin J Pain.* 1992;8(2):77-85 | Rarely addictive | 196 |
| 6 | Joranson DE, Ryan KM, Gilson AM, Dahl JL. Trends in medical use and abuse of opioid analgesics. *JAMA.* 2000;283(13):1710-1714. | Rarely addictive | 315 |
| 7 | Fishbain DA, Cole B, Lewis J, Rosomoff HL, Rosomoff RS. What percentage of chronic nonmalignant pain patients exposed to chronic opioid analgesic therapy develop abuse/addiction and/or aberrant drug-related behaviors? A structured evidence-based review. *Pain Med.* 2008;9(4):444-459 | Rarely addictive | 537 |
| 8 | Weissman DE, Haddox DJ. Opioid pseudoaddiction--an iatrogenic syndrome. *Pain.* 1989;36(3):363-366. | Pseudoaddiction | 254 |
| 9 | McQuay H. Opioids in pain management. *Lancet.* 1999;353(9171):2229-2232 | Pseudoaddiction | 143 |
| 10 | Fishbain DA. Medico-legal rounds: Medico-legal issues and breaches of "standards of medical care" in opioid tapering for alleged opioid addiction. *Pain Med.* 2002;3(2):135-142; discussion 143-136 | Pseudoaddiction | 1 |
| 11 | Portenoy RK, Foley KM, Inturrisi CE. The nature of opioid responsiveness and its implications for neuropathic pain: new hypotheses derived from studies of opioid infusions. *Pain.* 1990;43(3):273-286. | No dose too high | 95 |
| 12 | Foley KM, Fins JJ, Inturrisi CE. A true believer's flawed analysis. *Arch Intern Med.* 2011;171(9):867-868; author reply 868 | No dose too high | 10 (all study protocols) |
| 13 | Webster LR, Webster RM. Predicting aberrant behaviors in opioid-treated patients: preliminary validation of the Opioid Risk Tool. *Pain Med.* 2005;6(6):432-442. | Screening tools | 1175 |
| 14 | Belgrade MJ, Schamber CD, Lindgren BR. The DIRE score: predicting outcomes of opioid prescribing for chronic pain. *J Pain.* 2006;7(9):671-681. | Screening tools | 392 |
| 15 | Passik SD, Kirsh KL. An opioid screening instrument: long-term evaluation of the utility of the pain medication questionnaire by Holmes et al. *Pain Pract.* 2006;6(2):69-71. | Screening tools | 54 |

Abbreviations: OIDA, Opioid Industry Documents Archive

^a^ OIDA search conducted on July 20, 2023

**eFigure. Notable Documents from OIDA Citing Scientific Articles to Support Claims**

**Claim 1: Efficacy for Treatment of Chronic Non-Cancer Pain**

Declaration of Russell K. Portenoy, MD

**
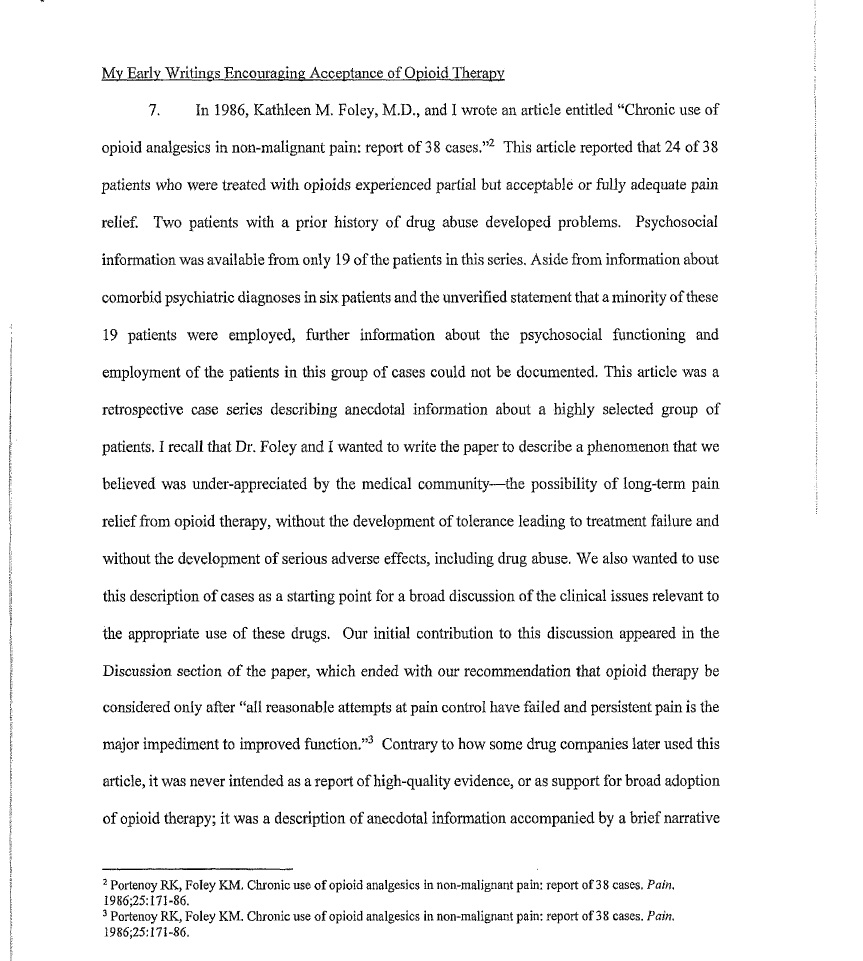
**

Statement from the lead author of the paper, Russel K. Portenoy, discussing the article itself, how he and his coauthor intended the study to be characterized, and how it was not meant to be used as high-quality evidence for the claim of opioid effectiveness for noncancer pain, as the opioid industry used it.

**Claim 2: Rarity of Addiction**

Opioids: The Good, Bad, and The Ugly


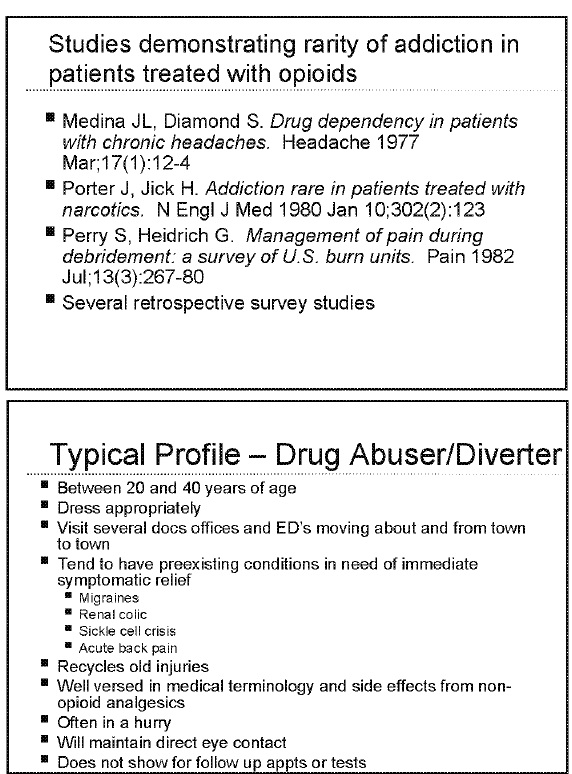


Presentation at Pain Weeks conference by Sean Mackey, the past president of American Academy of Pain Medicine. Relevant slides describing opioids and addiction. Cited Perry et al and Porter et al.

Clinical Evaluation of the Patient with Chronic Pain


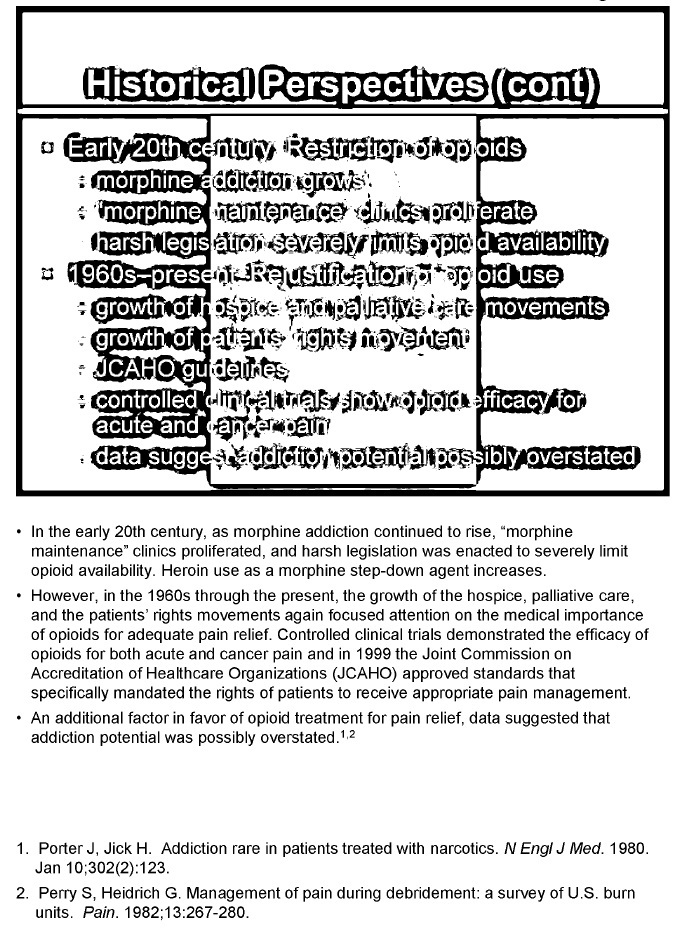


2019 presentation by the Director of Cohn Pain Management Center at North-Shore-Long Island Jewish Health System entitled, "Clinical Evaluation of the Patient with Chronic Pain." Includes slide that says, "data suggested addiction possibly overstated" and cited both Perry and Porter articles.

A Pharmacist’s Guide to Counseling Patients on the Role of Opioid Analgesics in Pain Management


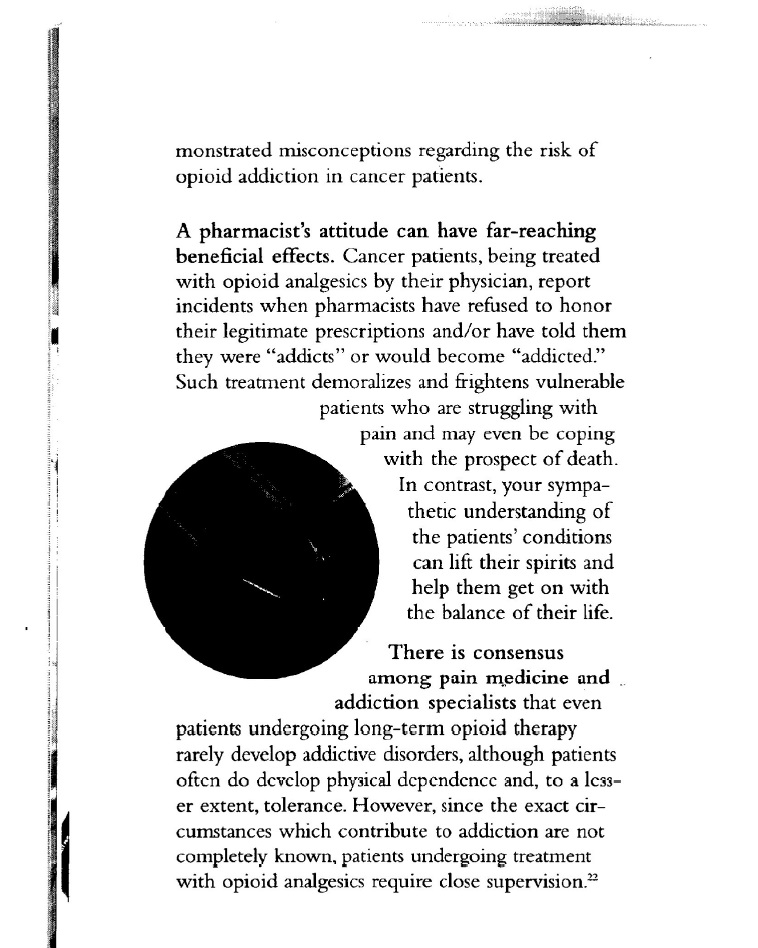


Pharmacist educational material entitled, "A Pharmacist's Guide to Counseling Patients" on how to counsel patients being treated with opioids for pain based on a misrepresentation of the study.

Counseling Your Patients and Their Families Regarding the Use of Opioids to Relieve Pain


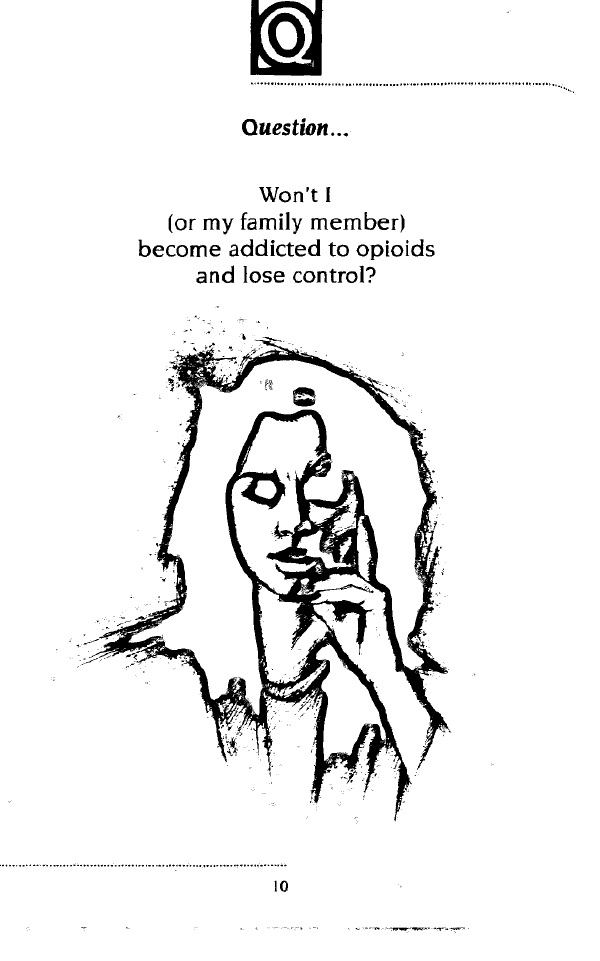

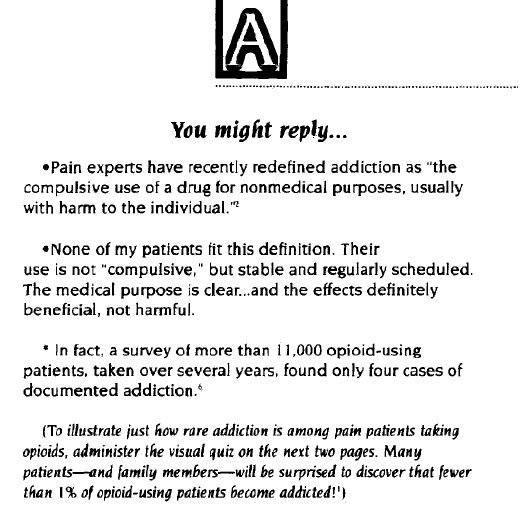


Purdue-backed group, Partners Against Pain, teaching front-liners/doctors how to answer patient questions, including, "Won't I (or my family member) become addicted to opioids and lose control?" They cite the paper in response, "In fact, a survey of more than 11,000 opioid-using patients, taken over several years, found only four cases of documented addiction."

Substance Abuse Issues in Pain Management, Steven D. Passik, MD


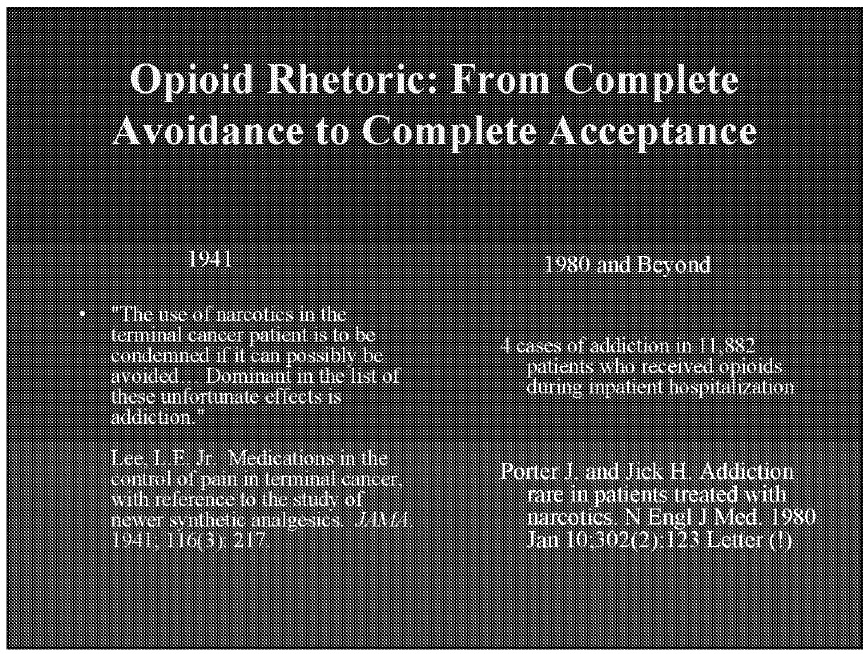


Presentation by Passik using the Porter article to explain the transition "From Complete Avoidance to Complete Acceptance".

Opioid analgesics - advanced sales training


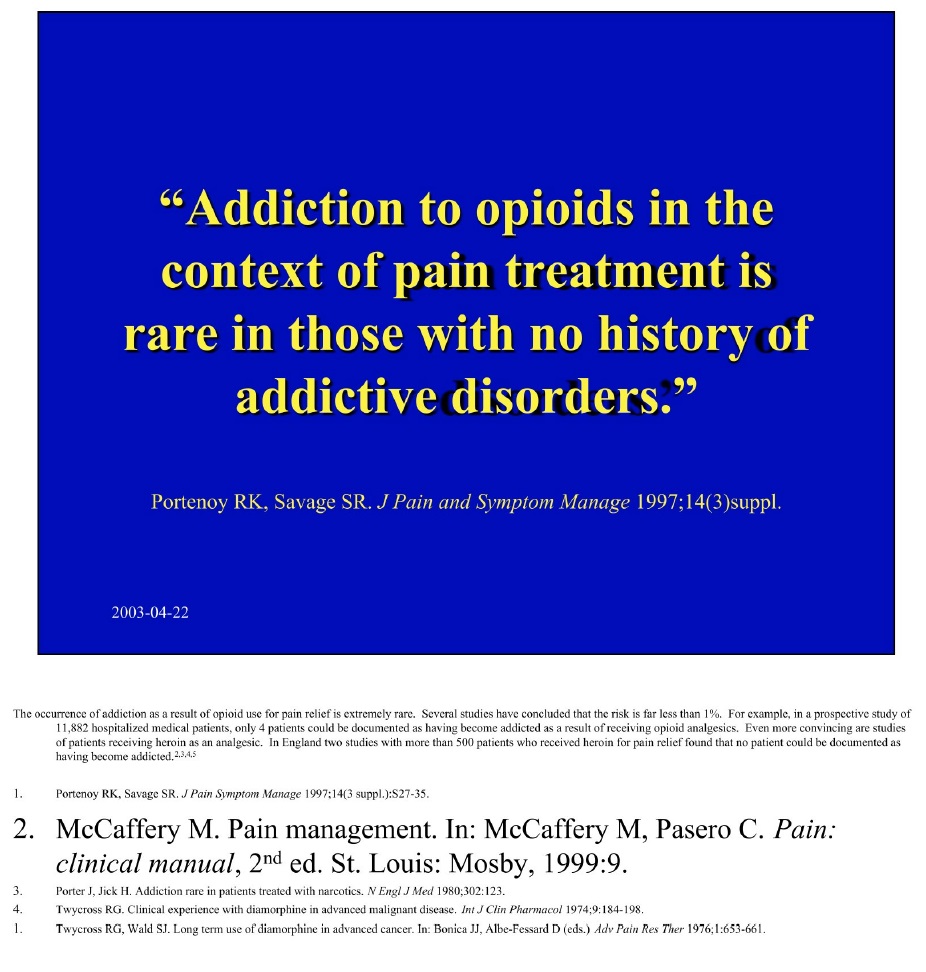


From a sales training, presentation slide citing Porter et al’s findings.

Optimizing Chronic Pain Management with Duragesic Fentanyl Transdermal System


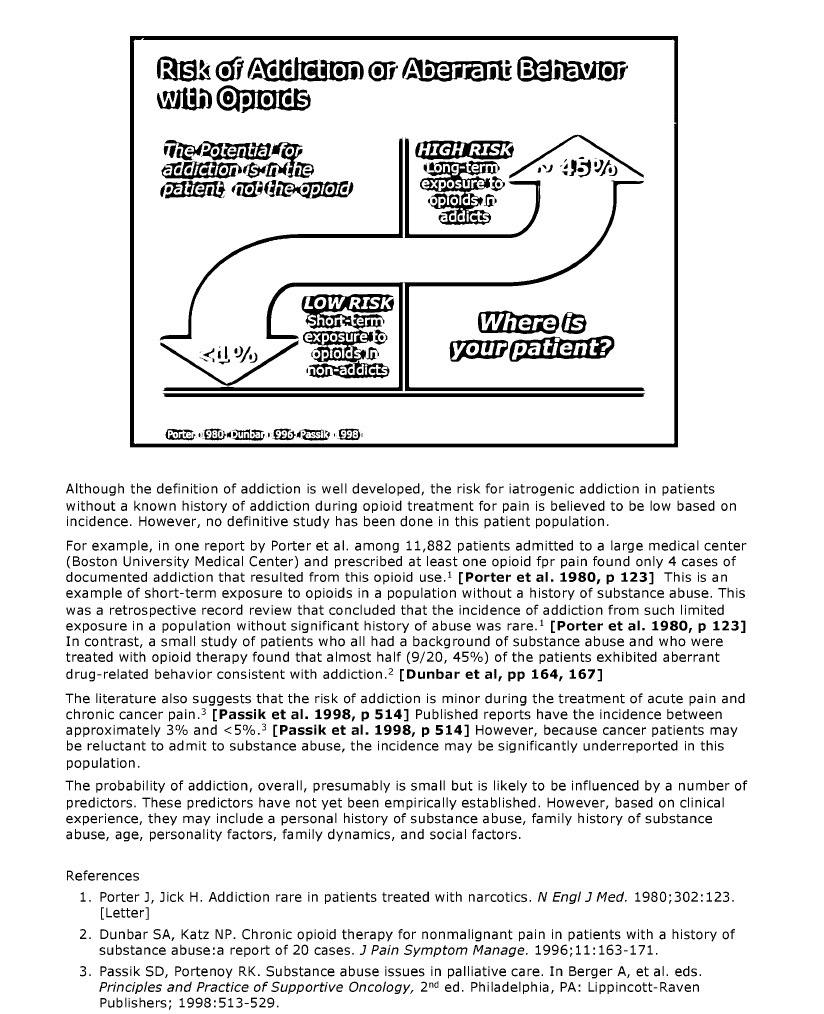


Educational resource for unclear audience (physicians?) provided by Janssen Pharmaceutical Products, L.P." States, “The probability of addiction, overall, presumably is small but is likely to be influenced by a number of predictors.” Cited Porter et al.

Summary/Conclusions: Pain Management Speakers Training Program for Nurses and Pharmacists


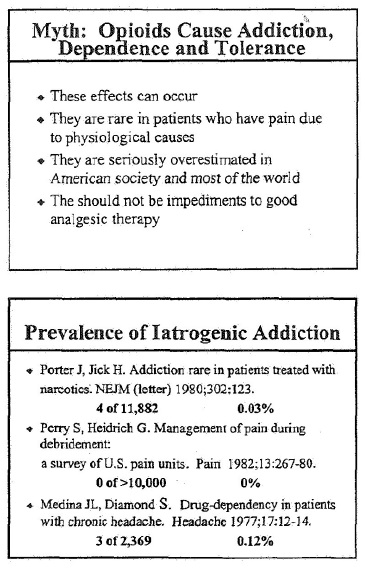


Pain Management Speakers Training Program for Nurses and Pharmacists in 2000. Two slides included that claimed it was a myth that "opioids cause addiction, dependence and tolerance." Cited Porter et al and Perry et al.

Email from Medical Services, Product Management, Sales Training, Janssen Pharmaceuticals to Sales Force


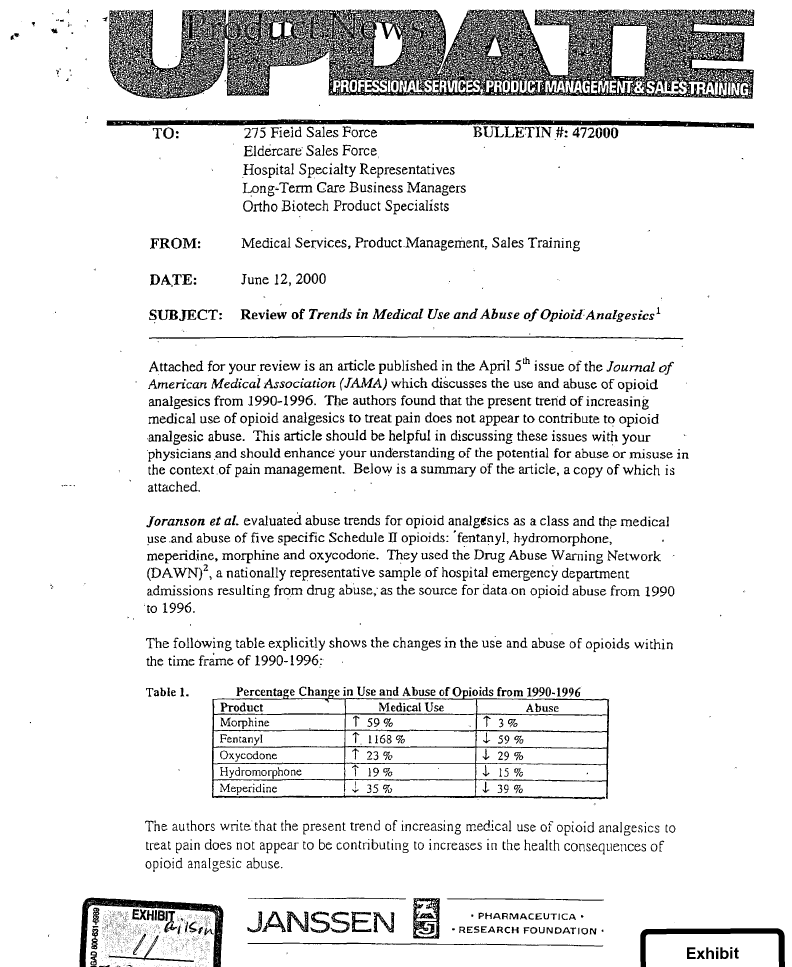


2000 Email to Eldercare Sales Force from Medical Services, Product Management, Sales Training at Janssen Pharmaceuticals alerting them about the publication of the Joranson article, describing the study, and stating, "The authors found that the present trend of increasing medical use of opioid analgesics to treat pain does not appear to contribute to opioid analgesic abuse. This article should be helpful in discussing these issues with your physicians and should enhance your understanding of the potential for abuse or misuse in the context of pain management."

Email from Pain Management Consultant and Educator to Editor of Portland Press Herald


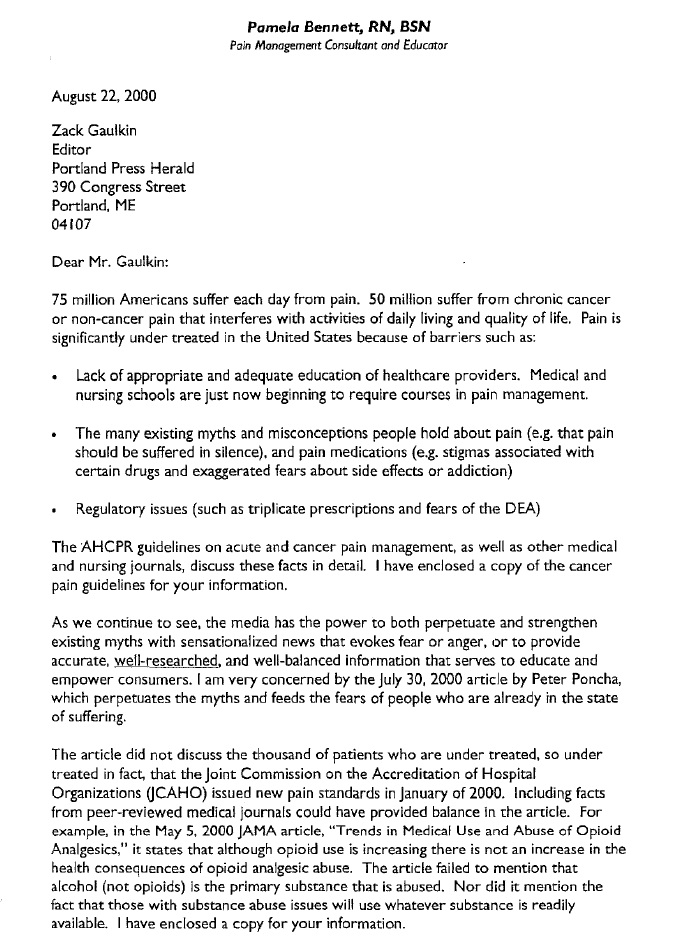


2000 email from Pamela Bennett to the editor of Portland Herald Press using Joranson article to say that abuse is low. She eventually went to go work for Purdue (https://www.emedevents.com/speaker-profile/pamela-bennett)

**Claim 3: “Pseudoaddiction”**

Opioid analgesics - advanced sales training

**
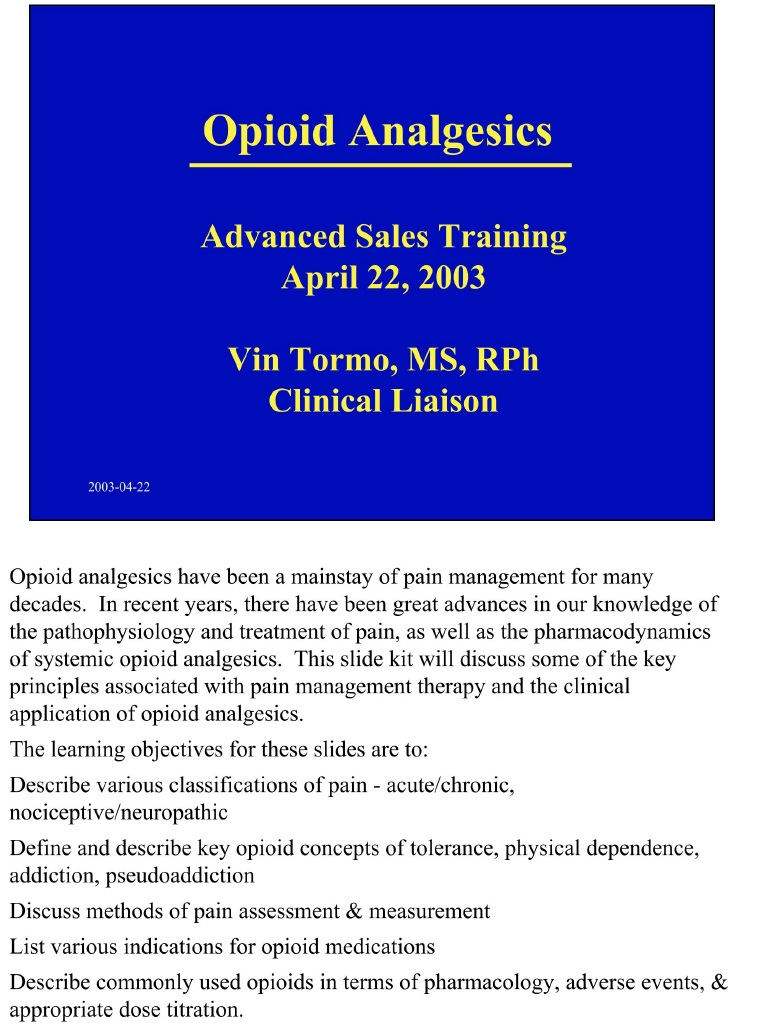

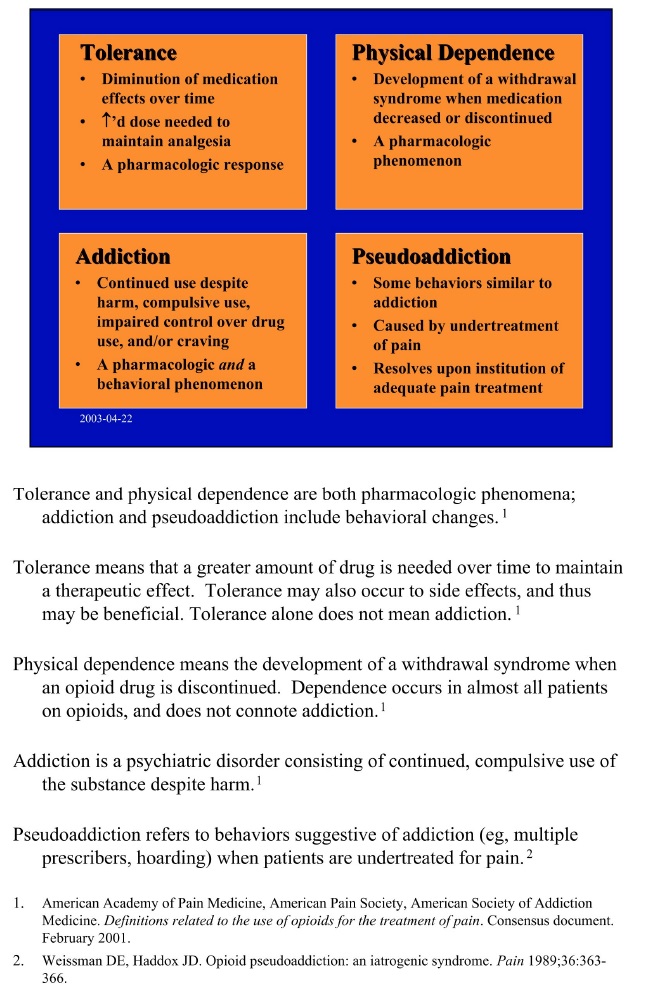
**

From a sales training, the title slide and a presentation slide describing the concept of “pseudoaddiction.”

Exalgo MSL Slide Deck Outline - 10.26.09

**
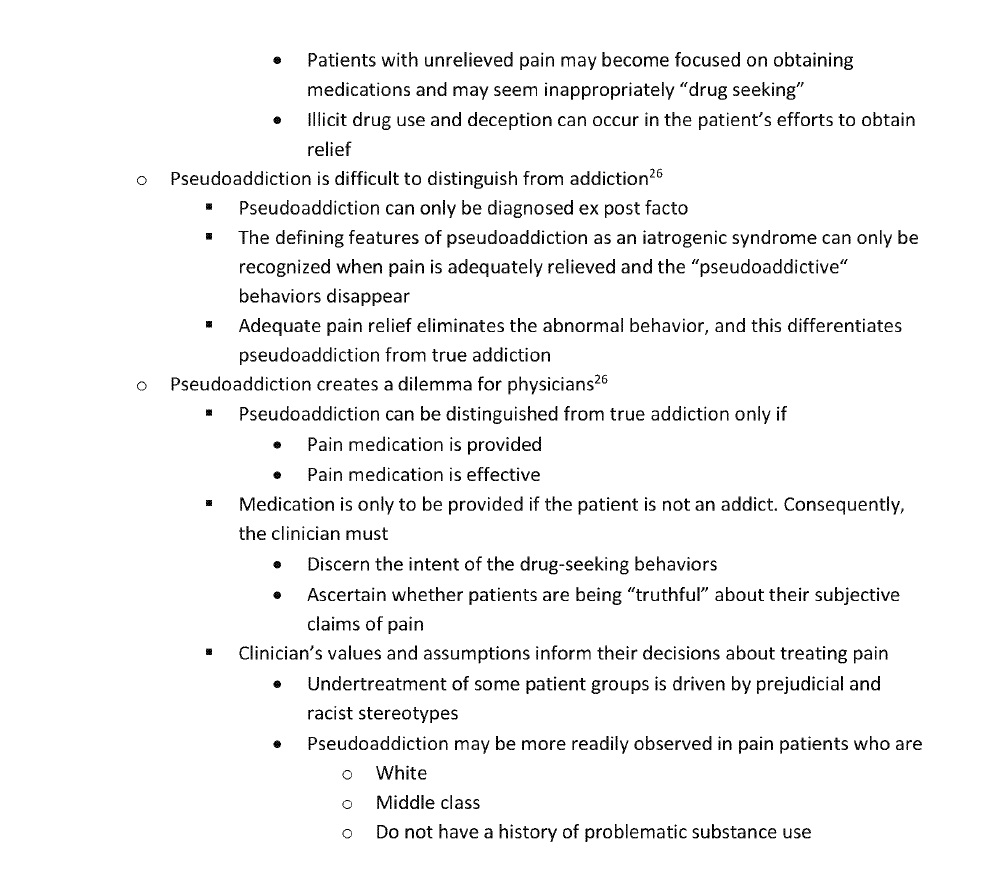
**

Notes for a presentation from Covidien to medical science liaisons. Included “pseudoaddiction” as a consideration for using opioids for chronic pain, expanding on the concept beyond what the Weissman paper stated.

**Claim 4: No Dose is Too High**

Advances in Pain Management^a^


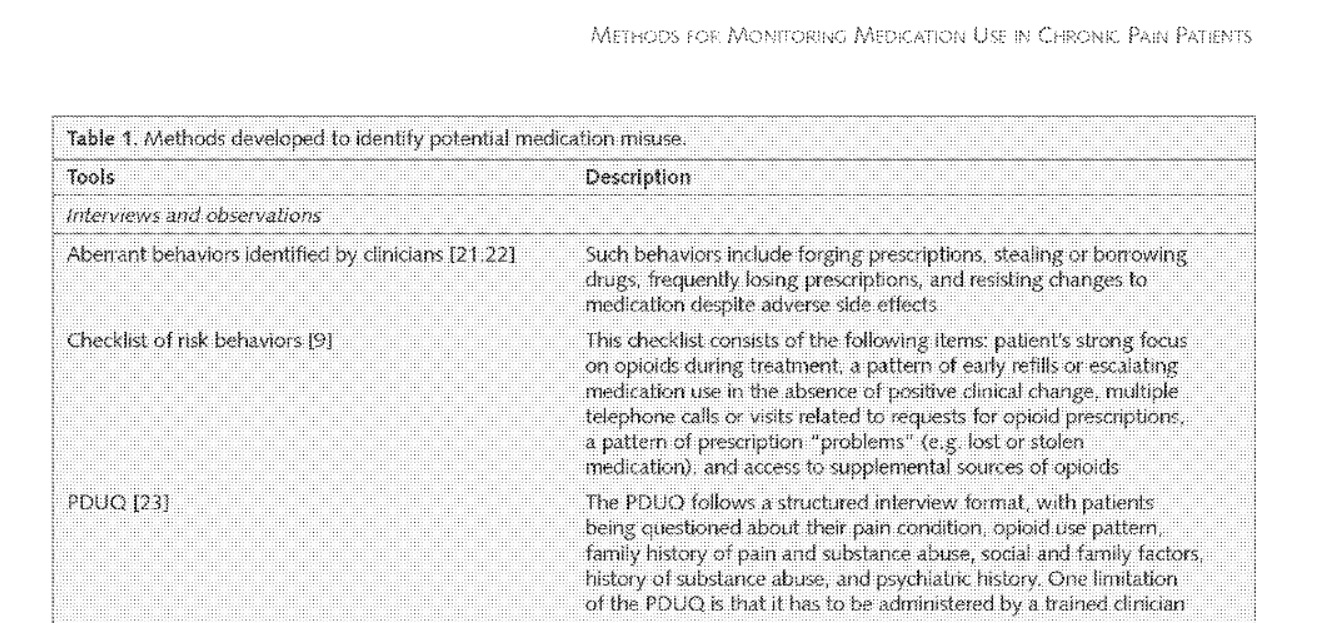


Table from a review article included in a 2008 continued medical education activity jointly sponsored by the University of Kentucky and an educational grant from the pharmaceutical company Cephalon. The Portenoy 1990 study was citation #22. Portenoy was an associate editor of Advances in Pain Management, an independent journal in which the CME activity was published.

**Claim 5: Screening Tools Can Predict Addiction Risk**

Protect Your Patients, Protect Your Practice – Practical risk assessment in the structuring of opioid therapy in chronic pain
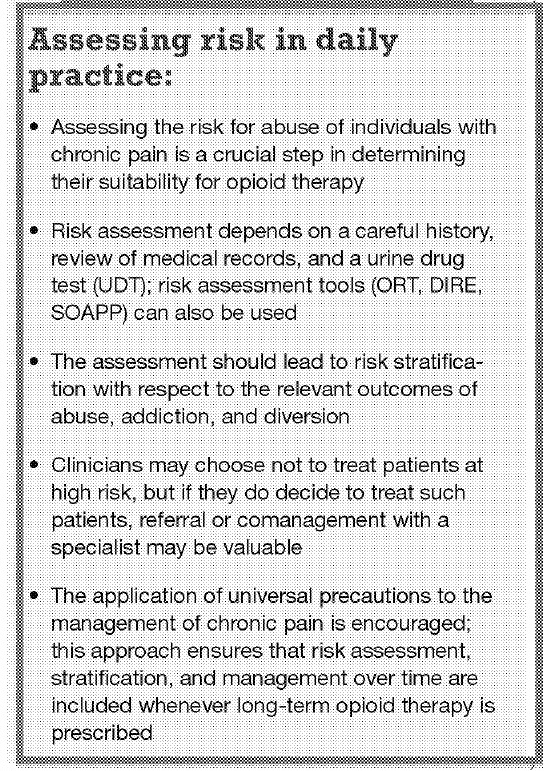


Exhibit from a Continued Medical Education Activity, a Supplement to The Journal of Family Practice, supported by educational grants from Endo Pharmaceuticals and Ortho-McNeil-Janssen Pharmaceuticals, with Portenoy as the Chair of the Steering Committee. The authors state that risk assessment tools like DIRE can be used for "assessing risk in daily practice". Cited Belgrade 2006.

Navigating REMS in Opioid-Tolerant Patients: An Interactive Workshop


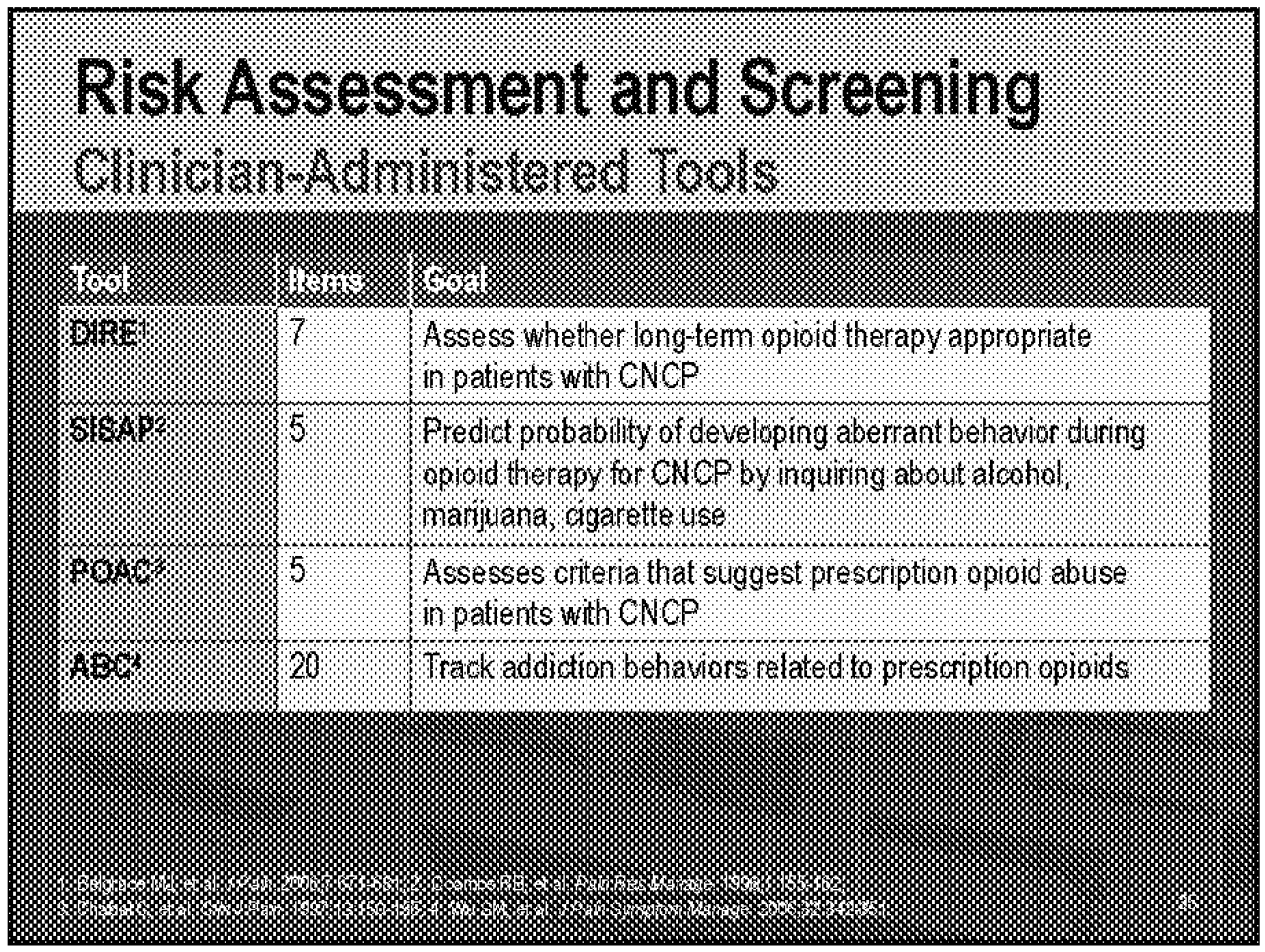


Continued medical education interactive workshop as part of REMEDIES: Focus on Opioid Tolerance, sponsored by Global Education Group and Applied Clinical Education and supported by an educational grant from Mallinckrodt and Covidien. Cited Belgrade 2006.

Case Challenges in Chronic Pain: Integrating Opioid Therapy with Multimodal Interventions to Optimize Outcomes


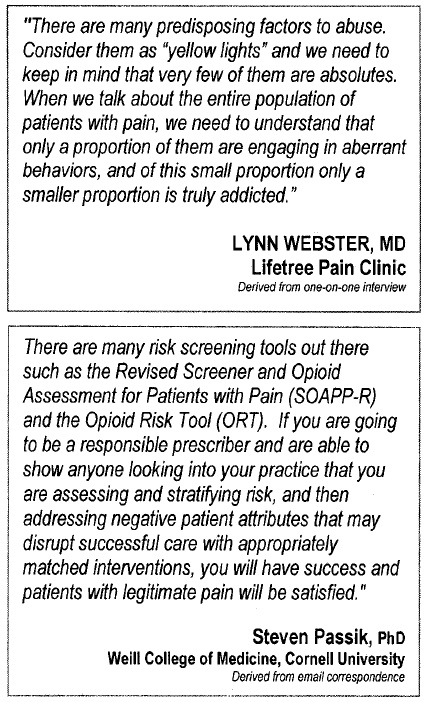


2012 continued medical education activity submitted by Penn State University College of Medicine at the 2012 Annual Scientific Meeting of the American Pain Society, entitled, "Case Challenges in Chronic Pain: Integrating Opioid Therapy with Multimodal Interventions to Optimize Outcomes." Cited Webster, 2005.

Opioids in Acute Pain Management


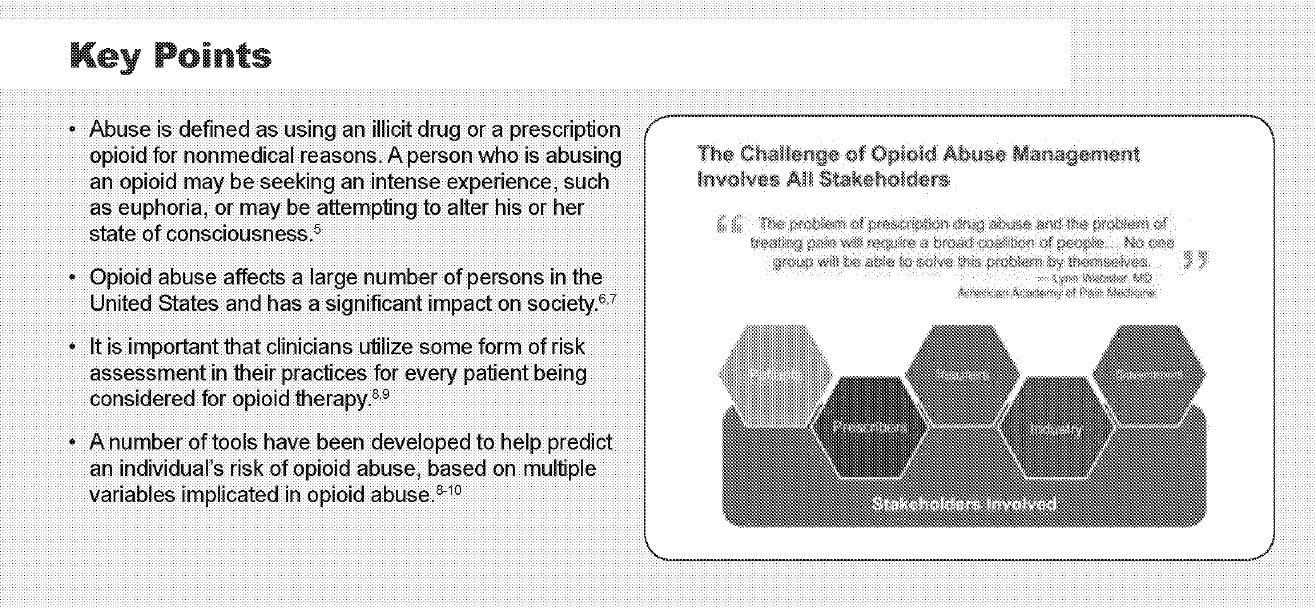


Presentation entitled "Opioids in Acute Pain Management" by Mallinckrodt Pharmaceuticals. Citation #10 is Webster 2005.

Covidien Advisory Council – For Responsible Prescribing and Safe Use


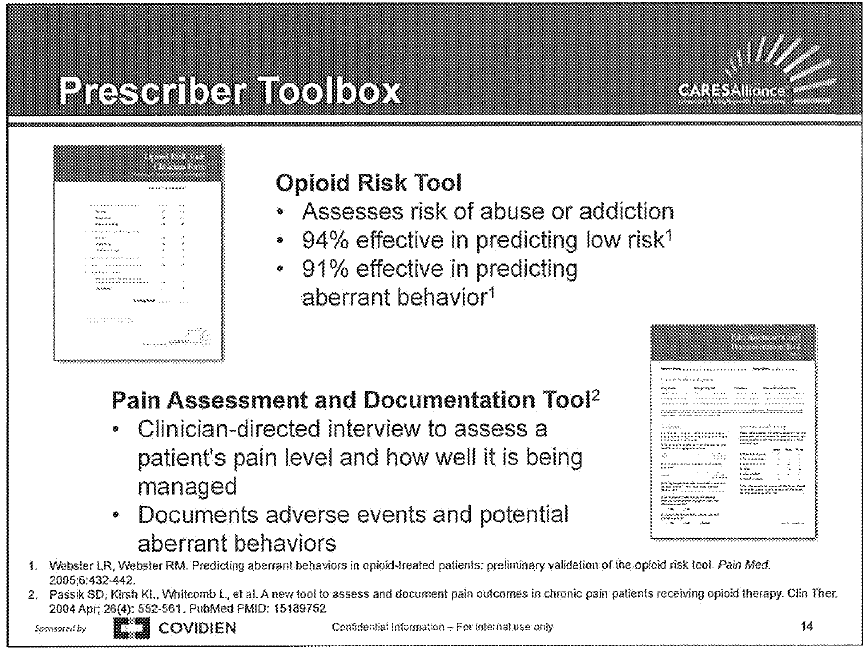


In an effort to mitigate risk of opioids, discussed the role of tools to identify the right patient for opioids and cited the opioid risk tool.

FW: New Nucynta ER ePromotion - Just In From The CDP Panel


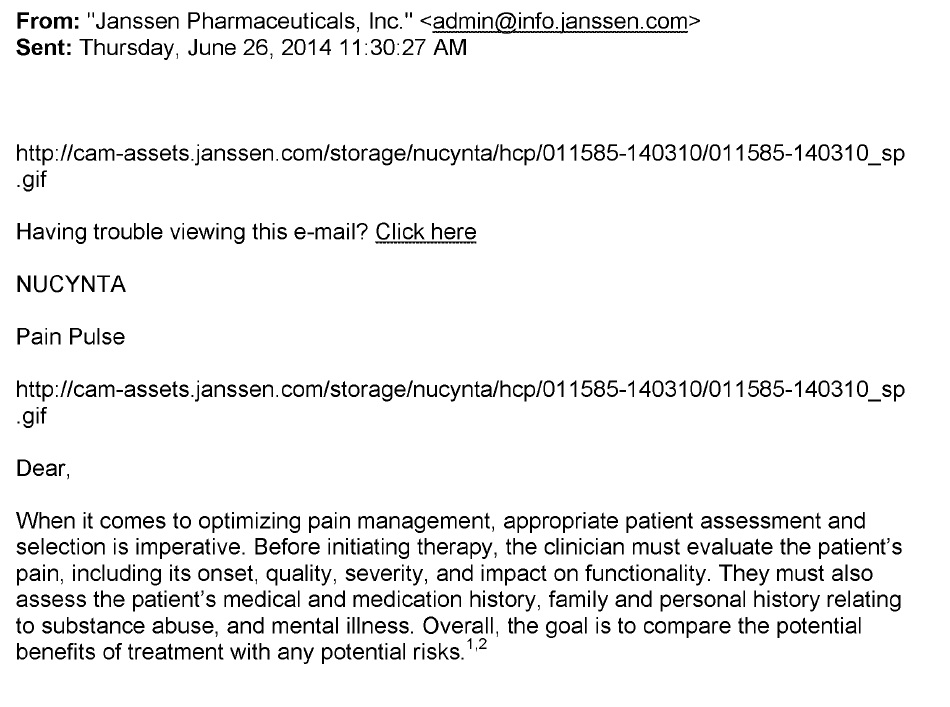

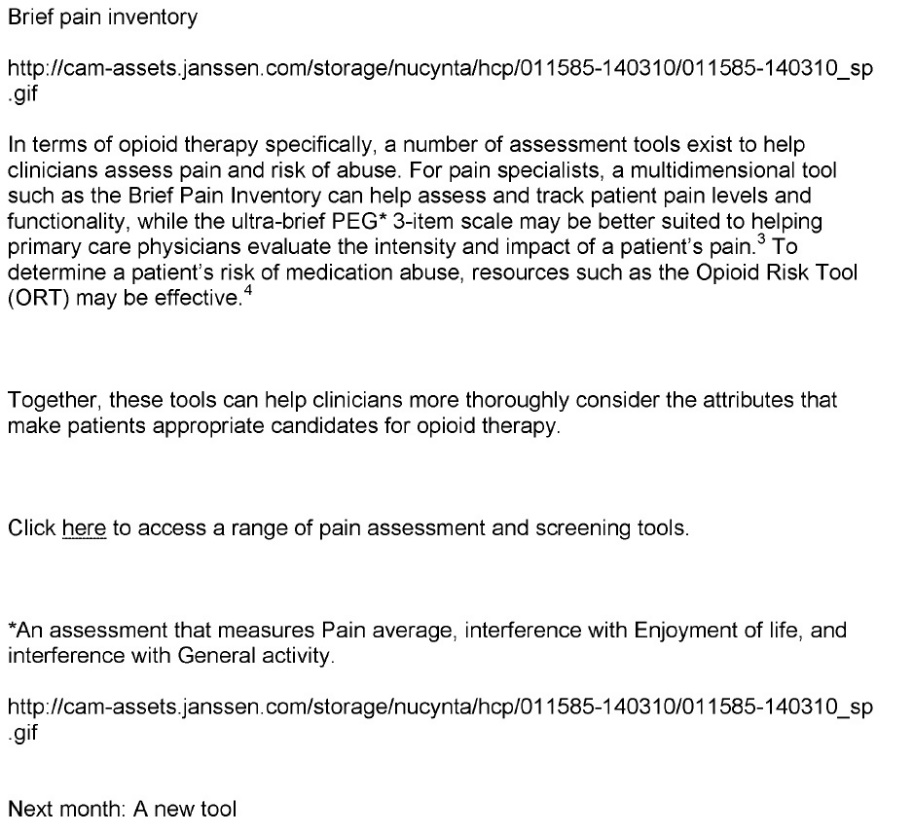


2014 promotional email from Janssen Pharmaceuticals for Nucynta targeting physicians.

Assessment of ESP Online CME Activities for Pain Physicians


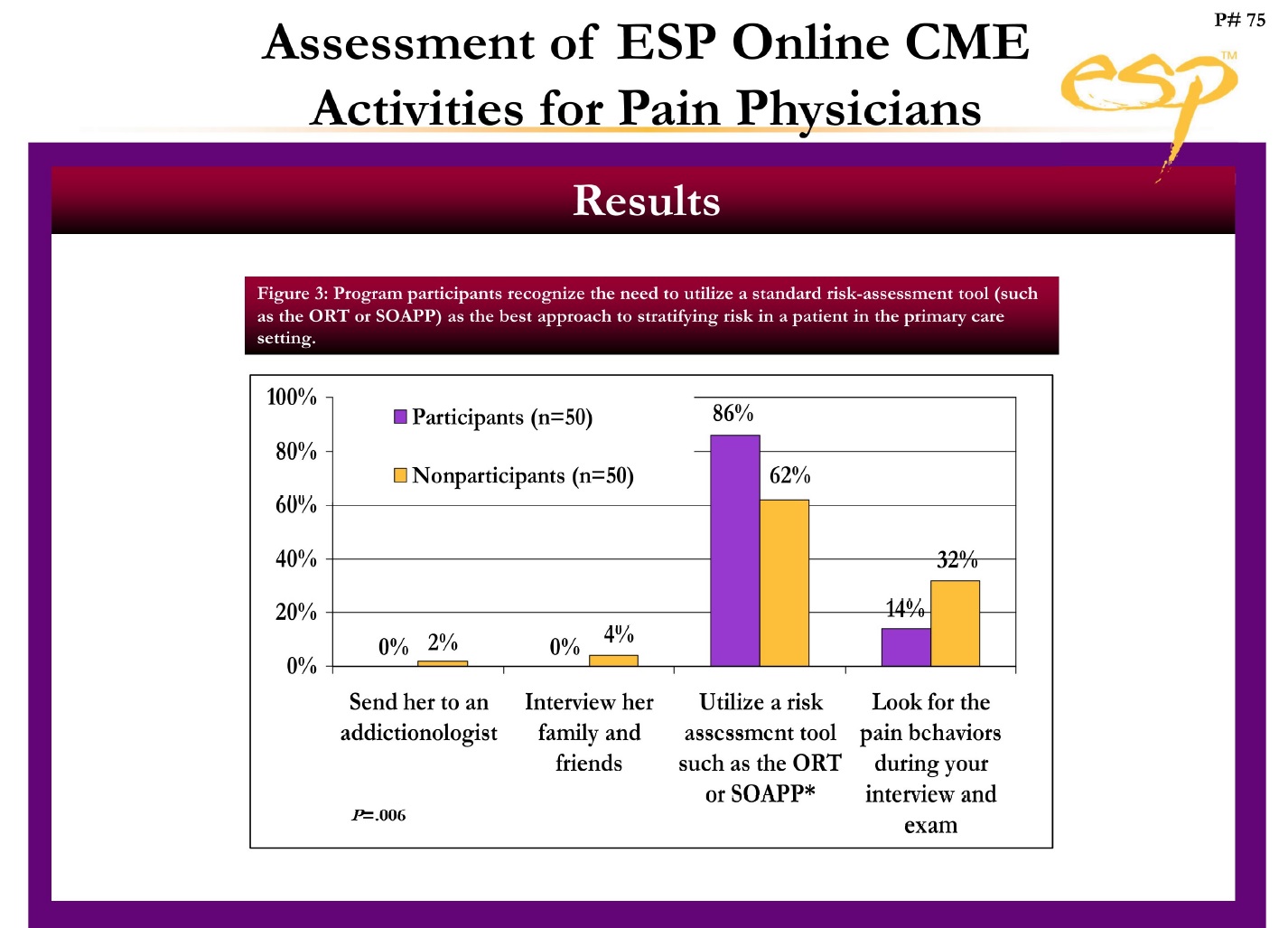


2010 email exchange between MediComWorldwide, Inc (provider of continued medical education) and Teva sharing Emerging Solutions in Pain Outcomes Assessment slides that were created by MediCom Worldwide and supported by an educational grant from Cephalon, Endo Pharmaceuticals, PriCara, a division of Ortho-McNeil-Janssen Pharmaceuticals, Inc., and Purdue Pharma, L.P. The CME activity was targeted towards primary care physicians and pain specialists. Found that providers engaging in the Emerging Solutions in Pain activity were more likely to utilize risk assessment tools such as the Opioid Risk Tool (ORT) or the Screener and Opioid Assessment for Patients with Pain (SOAPP)."
